# Characterization and drug sensitivity of a novel human ovarian clear cell carcinoma cell line genomically and phenotypically similar to the original tumor

**DOI:** 10.1002/cam4.1724

**Published:** 2018-08-14

**Authors:** Miriam Franklin, Lucy Gentles, Elizabeth Matheson, Nick Bown, Paul Cross, Angela Ralte, Connor Gilkes‐Immeson, Alice Bradbury, Maryam Zanjirband, John Lunec, Yvette Drew, Rachel O'Donnell, Nicola J. Curtin

**Affiliations:** ^1^ Northern Institute for Cancer Research Medical School Newcastle University Newcastle upon Tyne UK; ^2^ Division of Cancer Sciences School of Medical Sciences Faculty of Biology, Medicine and Health University of Manchester Manchester UK; ^3^ Northern Genetics Service Institute of Genetic Medicine Newcastle upon Tyne UK; ^4^ Pathology Department Queen Elizabeth Hospital Gateshead UK; ^5^ Department of Biology Faculty of Science University of Isfahan Isfahan Iran; ^6^ Northern Centre for Cancer Care Freeman Hospital Newcastle upon Tyne UK; ^7^ Northern Gynaecological Oncology Centre Queen Elizabeth Hospital Gateshead UK

**Keywords:** cytotoxic drugs, DNA repair, loss of heterozygosity, new cell line, NVP‐BEZ235, ovarian clear cell carcinoma, p53, PARP inhibitor

## Abstract

NUCOLL43 is a novel ovarian clear cell carcinoma (O‐CCC) cell line that arose from a primary culture of a patient's malignant ascites. The cells grow reliably in cell culture with a doubling time of approx. 45 hours and form colonies at high efficiency. They have a very high degree of loss of heterozygosity (LOH) affecting approximately 85% of the genome, mostly copy neutral and almost identical to the original tumor. The cells express epithelial (pan‐cytokeratin) and mesenchymal (vimentin) characteristics, CA125 and p16, like the original tumor. They also express ARID1A but not HNF‐1β and, like the original tumor, and are negative for p53 expression, with no evidence of p53 function. NUCOLL43 cells express all other DNA damage response proteins investigated and have functional homologous recombination DNA repair. They are insensitive to cisplatin, the PARP inhibitor rucaparib, and MDM2 inhibitors but are sensitive to camptothecin, paclitaxel, and NVP‐BEZ235. The NUCOLL43 cell line represents a distinct subtype of O‐CCC that is p53 and HNF‐1β null but expresses ARID1A. Its high degree of similarity with the original tumor genomically and proteomically, as well as the high level of LOH, make this an interesting cell line for O‐CCC research. It has been deposited with Ximbio.

## 1 INTRODUCTION

Ovarian cancer is the 7th most common cancer in women, with half the cases being in women under 65. Each year, there are around 240 000 newly diagnosed cases and >150 000 women die of ovarian cancer worldwide.^1^ Despite advances in surgery and chemotherapy, ovarian cancer has a relatively poor 5‐year survival rate of approximately 45% in developed countries. This is largely due to late stage at diagnosis, as a consequence of the lack of specific symptoms and the absence of reliable screening tests.^2^

There are a number of different histological subtypes of ovarian cancer, each with different etiologies, clinical presentations, and mortality rates, and each associated with distinct clusters of mutations.^3^ Despite the wide variability between these histological subtypes, they are currently all treated the same with cytoreductive surgery and platinum/taxane‐based chemotherapy.^4^ Nearly all of the most common subtype, high‐grade serous ovarian cancer (HGSC) has TP53 mutations, and just over half are associated with BRCA mutations or other defects in the BRCA‐associated homologous recombination DNA repair (HRR) pathway. These mutations underlie their sensitivity to platinum therapy, with initial response rates of 70%, and the recently introduced PARP inhibitors.^5^

Estimates of the frequency of clear cell carcinoma of the ovary (O‐CCC) vary substantially in the literature from 4%‐5% up to 26%.^6^ Variation may reflect differences in ethnicity and demographics of the populations sampled, sample size, and trends to increased diagnosis with time,^7^ which may also reflect trends in pathological diagnoses. The cell of origin for O‐CCC is believed to be endometrial, and endometriosis is a known risk factor for O‐CCC.^3^ Overexpression of TP53 (in its mutated inactive form) is seen in only 10%‐20% of O‐CCC cases, in contrast to the high incidence seen in HGSC.^8^ Generally, O‐CCCs respond poorly to chemotherapy with response rates of 15%‐45%, with the higher response rates possibly attributable to misdiagnosis of HGSC as O‐CCC.^6^ The high rates of resistance in O‐CCC highlight the need for further research to better understand the biology of this subset of ovarian cancer as well as therapies targeting their unique biology.^9^

Cell lines are the most commonly used model to study cancer in vitro. The numerous HGSC cell lines available were key in identifying BRCA/HRR pathway defects as major determinants of sensitivity to platinum‐based and PARP inhibitor chemotherapy for this subset of ovarian cancer. However, there are far fewer cell lines available that model the other subtypes of ovarian cancer. For O‐CCC, 27 cell lines have been described (Table S1), but most have not been extensively characterized and only 14 have been deposited in cell banks. Furthermore, recent research has highlighted cases of subtype misclassification of existing ovarian cancer cell lines.^10^, ^11^

The aim of this study was to characterize a novel cell line, designated NUCOLL43, which was derived from the ascitic fluid of a patient with clear cell adenocarcinoma of gynecological origin. NUCOLL43 cells grow well in culture with a consistent growth rate and stable phenotype. It is representative of its tumor of origin based on the pan‐genomic and phenotypic similarity to the original tumor. NUCOLL43 cells are resistant to cisplatin and the PARP inhibitor rucaparib, but sensitive to paclitaxel, camptothecin, and to NVP‐BEZ 235. Interestingly, the cell line shows complete loss of TP53 function and expressed protein despite no evidence of chromosome 17p deletion. It exhibits a high level of copy neutral loss of heterozygosity.

## 2 MATERIALS AND METHODS

### 2.1 Chemicals and reagents

All chemicals and reagents were obtained from Sigma (Poole, UK) unless otherwise stated. Camptothecin, paclitaxel, rucaparib (a gift from Pfizer Global R&D), VE‐821, NVP BEZ235 (Selleckchem), Nutlin‐3 (NewChem, Newcastle, UK) and RG7388 (kindly provided by Newcastle Anticancer Drug Development Initiative) were dissolved in dry dimethyl sulfoxide (DMSO) at concentrations of 1‐10 mmol/L and stored as aliquots at −20°C. Cisplatin was dissolved in PBS at 1 mmol/L and stored in aliquots at −20°C.

### 2.2 Cell line establishment

The cell line described in this study, NUCOLL43, was established from the ascitic fluid of a 57‐year‐old white British patient who presented with nonspecific symptoms of abdominal bloating and distension. Subsequent investigations demonstrated marginally elevated serum cancer antigen 125 (CA125) of 110 U/mL, and CT imaging showed ovarian masses with disseminated intra‐abdominal metastases consistent with advanced stage ovarian cancer (FIGO stage 3C). She underwent a diagnostic laparotomy to assess for cytoreductive surgery; however, complete cytoreduction was not possible due to the extensive nature of her disease. Following drainage of ascites, a biopsy was taken, which confirmed CCC of gynecological origin. The patient unfortunately deteriorated with progressive disease, was not fit enough to undergo chemotherapy and died 6 weeks later. Ethical approval and written consent were obtained for the collection of clinical material and patient data (REC 12/NW/0202, REC 12/NE/0395). The aspirated ascites collected from the patient were cultured ex vivo as previously described.^4^ Following a period of apparent senescence at around 5 weeks, colonies of cells began to appear approximately 2 weeks later (Figure S1). From this point, the cells were cultured in RPMI‐1640 medium supplemented with 20% fetal bovine serum (FBS) (Gibco) and grown at 37°C in a 5% CO₂ humidified atmosphere. For all experiments, the cells used were in their exponential growth phase.

### 2.3 Pathology and immunohistochemistry

A portion of the tumor was removed during surgery and fixed in 10% buffered formalin. The formalin fixed paraffin embedded (FFPE) tissue was cut into four micron thick sections and taken on TOMO® Adhesive Microscope Slides. Immunohistochemical staining was performed using the streptavidin‐avidin‐biotin immunoperoxidase method. All staining procedures were performed using the fully automated and fully integrated Ventana BenchMark ULTRA immunohistochemistry platform (© 2013 Ventana Medical Systems, Inc.). All antibodies used were supplied as ready to use, prediluted kits by Ventana Medical Systems (including cytokeratin, vimentin, p16, p53 clone DO‐7, Estrogen receptor clone SP‐1, CA125 OC125, PAX 8 MRQ‐50, Napsin A clone MRQ60). Immunostained sections were analyzed with Olympus System Microscope BX43 and Leica DM2500 microscopes.

### 2.4 Genetic analysis

Karyotyping and SNP array analysis was carried out by the Northern Genetics Service. For SNP array analysis, DNA was extracted using a Qiagen QIAamp DNA Kit and symphony robot (NUCOLL43 cells and whole blood) or EZ1 robot (tumor) according to the manufacturer's instructions. DNAs were hybridized to whole genome Infinium CytoSNP‐850K BeadChip arrays (Illumina, San Diego, CA, USA) and results were analyzed using BlueFuse Multi v4.4 software (Illumina).

### 2.5 Short tandem repeat (STR) analysis

STR profiling was performed by NewGene Limited following extraction of DNA from frozen cell pellets alongside appropriate positive and negative controls. Eight short tandem repeat (STR) loci, plus Amelogenin, were amplified using the GenePrint® 10 System, supplied by Promega (Madison, WI, USA). The reaction products were processed using an Applied Biosystems® 3130× l Genetic Analyzer and the resulting data interpreted using GeneMarker® v2.6.0 software (SoftGenetics LLC, State College, PA, USA).

### 2.6 Western blot analysis

For analysis of p53 function, whole cell lysates were prepared from exponentially growing cells in lysis buffer (62.5 mmol/L Tris‐HCl pH 6.8, 2% w/v SDS), 10% v/v Glycerol) and then sonicated. For all other proteins, exponentially growing cells were lysed with RIPA buffer with 1:100 protease inhibitor cocktail (Thermo Fisher Scientific) or Phosphosafe extraction reagent (Merck) following the manufacturer's protocol. The protein content of all lysates was determined by Pierce BCA assays (Thermo Fisher Scientific, Waltham, MA, USA, Waltham, MA, USA) according to the manufacturer's protocol. For measurement of DNA damage response (DDR), proteins samples were diluted in XT sample buffer (Bio‐Rad, Hemel Hempstead, UK) and XT reducing agent (Bio‐Rad) and 30 μg protein was loaded to each lane of a 3‐8% Criterion XT Tris‐acetate gel (Bio‐Rad) for electrophoresis. For analysis of p53 function, Novex® 4‐20% Tris‐glycine 12‐well polyacrylamide gradient gels (Invitrogen, UK) were used. For all other proteins samples, diluted in 2x Laemmli buffer (Bio‐Rad) and deionized water, were denatured by heating to 95°C for 5 minutes. 20‐25 μg of protein was loaded per lane into a 4‐20% Criterion TGX polyacrylamide gel (Bio‐Rad) for electrophoresis. The separated proteins were transferred to nitrocellulose Hybond™ C membranes (Amersham, Buckinghamshire, UK). Membranes were blocked with 5% milk (w/v) or 5% BSA (w/v) (for DDR proteins) in TBST, before incubation with primary antibodies in the same buffer: mouse anti‐MDM2 (1:300 Merck Millipore #: OP46‐100UG), mouse anti‐p21^WAF1^ 1:100 (#: OP64, Calbiochem), mouse anti‐p53 (1:500 Leica Microsystems Ltd. #: NCL‐L‐p53‐DO7), rabbit anti‐HNF‐1β (1:400, Sigma #: HPA002083), mouse anti‐p16 (1:1000, Abcam #: DCS50.1), mouse anti‐ER‐α (1:200, Santa Cruz #: D‐12), rabbit anti‐ARID1A (1:1000, Cell Signaling #: 12354), rabbit anti‐ATM (1:500, Cell Signaling #: 2873S), goat anti‐ATR (1:500, Santa Cruz #: 1887), rabbit anti‐BRCA1 (1:500, Abcam #: 47573), rabbit anti‐DNA‐PKcs (1:500, Santa Cruz #: 9051), mouse anti‐Ku70 (1:500, Abcam #: Ab80592), rabbit anti‐Ku80 (1:500, Abcam #: Ab3114), PARP1 (1:500, Biovision #: 3001‐100), rabbit anti‐RAD51 (1:100, Santa Cruz #: 8349), rabbit anti‐XRCC1 (1:500, Santa Cruz #: 11429), mouse antiactin (1:1000, Sigma‐Aldrich #: A4700), mouse anti‐α‐tubulin (1:20 000, Sigma #: T6074) and rabbit antivinculin (1:1000, Cell Signaling #: 13901). The membranes were then incubated with HRP‐conjugated secondary anti‐mouse (Dako #: P0448), anti‐rabbit (Dako #: P0448) or anti‐goat (Santa Cruz #:2020) IgG antibodies were used at 1:2000. Clarity Western enhanced chemoluminescence substrate (Bio‐Rad) was used to visualize the bands following the manufacturer's protocol, and luminescence measured using G‐box gel documentation system (Syngene). For analysis of p53 function, X‐ray film (Fujifilm) was used to visualize the proteins.

### 2.7 Immunofluorescence microscopy

Cells were seeded onto sterile coverslips and allowed to adhere and grow to 80% confluence prior to fixation with ice‐cold methanol. Coverslips were washed with potassium chloride (KCl) buffer (120 mmol/L KCl, 20 mmol/L NaCl, 10 mmol/L Tris‐HCl, 1 mmol/L EDTA plus 0.2% Triton X‐100), blocked with 2% bovine serum albumin (BSA) diluted in KCl buffer, and incubated overnight at 4°C with primary antibodies: mouse anti‐pan‐cytokeratin (1:300, Abcam #: PCK‐26), rabbit antivimentin (1:250, Abcam #: EPR3776), mouse anti‐P16 (1:500, Abcam #: DCS 50.1) or rabbit anti‐CA125 (1:500, Epitomics #: EPR1020(20). After washing with KCl buffer, coverslips were incubated with corresponding fluorescently labeled secondary antibodies (Alexa Fluor 488 goat anti‐rabbit, Alexa Fluor 546 goat anti‐mouse (Invitrogen Life Technologies, Carlsbad, CA, USA)) diluted 1:1000 in 2% BSA (w/v) KCl buffer. After washing with KCl, the coverslips were mounted onto slides using VectaShield with DAPI (Vector Laboratories, Peterborough, UK) and viewed immediately using a Leica DMR fluorescence microscope.

### 2.8 Analysis of p53 function

The function of p53 was determined by measuring its induction and stabilization following 6 hours exposure to 0.5 μmol/L of the MDM2 inhibitor RG7388 (Idasanutlin) and consequent induction of MDM2 and p21 protein expression by Western blot as described above. The sensitivity of NUCOLL‐43 to the MDM2 inhibitors, Nutlin‐3a and RG7338 was determined as previously described.^12^

### 2.9 Homologous recombination DNA repair (HRR) assay

The HRR status of the cells was assessed as previously described.^13^ Briefly, cells were seeded onto glass coverslips and untreated or irradiated (2 Gy) and treated with rucaparib (10 μmol/L) for 24 hours to collapse replication forks then fixed in ice‐cold methanol. Coverslips were blocked with KCl buffer containing 2% BSA (w/v), 10% skimmed milk powder (w/v) and 10% goats serum (v/v) incubated overnight at 4°C with anti‐RAD51 antibody (1:500, Abcam #: ab133534) then anti‐phospho‐Histone H2A.X (Ser139) (1:1000 Merck #: JBW301) for 1 hour. Following incubation with Alexa Fluor secondary antibodies, mounting and microscopy, as above, the number of γH2AX and RAD51 foci/nucleus were counted using ImageJ software with the PZFociEZ macro (http://www.pzfociez.com). Cells were classified HRR competent if there was a ≥2‐fold increase in RAD51 foci after DNA damage, confirmed by a ≥2‐fold increase in γH2AX.

### 2.10 Proliferation assay

Cell proliferation was assessed by measurement of protein content of the population by sulforhodamine B (SRB) assay as previously described.^14^ Briefly, cells were seeded at densities of between 50 and 5000 cells/well and fixed sequentially over a 10‐day period. They were stained with 0.4% SRB and cell density estimated at 570 nm using a 96‐well plate spectrophotometer (SpectraMax 250 Molecular Devices). Cell doubling times were calculated for cells undergoing exponential growth using GraphPad Prism software (San Diego, CA, USA).

### 2.11 Cytotoxicity assays

Colony formation assays were used to determine the cytotoxic effect of various drugs on the cells, as previously described.^15^ Briefly, cells were seeded at densities of 50‐10 000 cells per well in six well plates. After 24 hours, the cells were incubated with varying concentrations of a chemotherapeutic agent (camptothecin 0‐100 nmol/L, rucaparib 0‐100 μmol/L, paclitaxel 0‐100 nmol/L, cisplatin 0‐10 μmol/L (in the presence and absence of 1 μmol/L VE‐821), NVP‐BEZ235 0‐300 nmol/L) for 24 hours with a final DMSO concentration of 0.5%, before being returned to drug‐free media. For rucaparib radiopotentiation studies, cells were seeded as above and irradiated (0‐8 Gy) in the presence and absence of 1 μmol/L rucaparib and returned to drug‐free medium 24 hours later. After 7‐day incubation, cells were fixed with Carnoy's fixative (methanol: acetic acid, 3:1 v/v) and stained with 0.4% crystal violet. Colonies were counted and cell survival determined by reference to the number of cells seeded and normalized to survival of vehicle alone (0.5% DMSO) or rucaparib or VE‐821 alone treated control cells. LC_50_ (concentration causing 50% cell death) values were determined by fitting a point‐to‐point curve using GraphPad Prism, (San Diego, CA, USA) version 6.

## 3 RESULTS

### 3.1 Establishment in culture and morphology of the cell line

**Figure 1:**
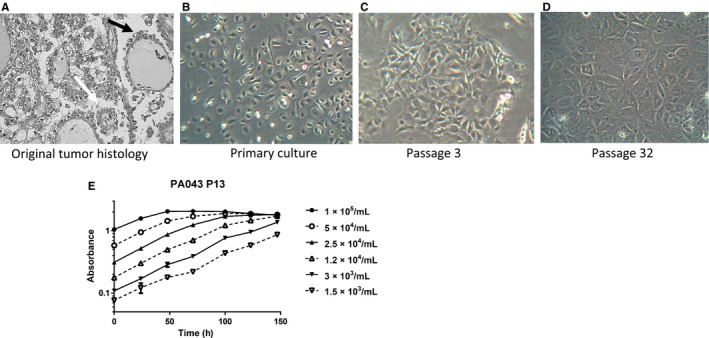
Morphology of the original tumor and NUCOLL43 and growth characteristics. H&E (x400) staining of the original tumor showing clear cells and hobnail cells (white arrow and black arrow, respectively: (A) and phase‐contrast microscopy (x400) of NUCOLL43 cells in primary culture (B) and at passages 3 (C) and 32 (D). Cells have epithelial morphology with prominent nucleoli and grow as confluent monolayers with a modest change of morphology from primary culture that is then maintained over 30 passages. Cells grew exponentially with time as determined by SRB staining until they reached confluence (Absorbance ≈ 2) (E)

The morphology of the original tumor is shown in Figure 1A (HE X50), where clear cells are visible, indicative of O‐CCC. The ascites were seeded in tissue culture flasks in 50% ascitic fluid, 50% RPMI + 20% FBS. After 15 days, the medium was replaced with fresh RPMI + 20% FBS at which stage they had typical primary epithelial culture morphology (Figure 1B). They underwent their first passage on day 23, and their epithelial nature was confirmed by pan‐cytokeratin staining, with a second passage 10 days later. Initially, the cells appeared to senesce but approximately 8 days later colonies began to appear (Figure S1) and the cells were successfully passaged repeatedly over several months. They exhibited the typical morphology of epithelial cell cultures (confirmed by pan‐cytokeratin staining at passage 1, 7 and 34 Figures S1 and 3) with multiple prominent nucleoli and grew to confluent monolayers with a cobblestone appearance (Figure 1C,D).

The proliferation of NUCOLL43 was determined by SRB assay. The doubling times of cells seeded at different densities varied from 33 to 52 hours (Figure 1E) and the mean doubling time of the cells at passages 13 and 35 was 45 and 42 hours, respectively, with no significant difference between the two passages. STR profiling on NUCOLL43 was undertaken at passage 22 and 35 with identical results each time (TH01: 8, TPOX: 11, vWA: 16, CSF1PO: 13, D16S539: 10, 12, D7S820: 11, 13, D13S317: 11, D5S818: 13 and Amelogenin: X) and without a complex electropherogram, indicating the cell line was not a mixture and was stable. NUCOLL43 was able to form colonies with high efficiency (approximately 50% plating efficiency) within 2 weeks.

### 3.2 Pan‐genomic and Chromosomal analysis of NUCOLL43 and the original tumor

**Figure 2:**
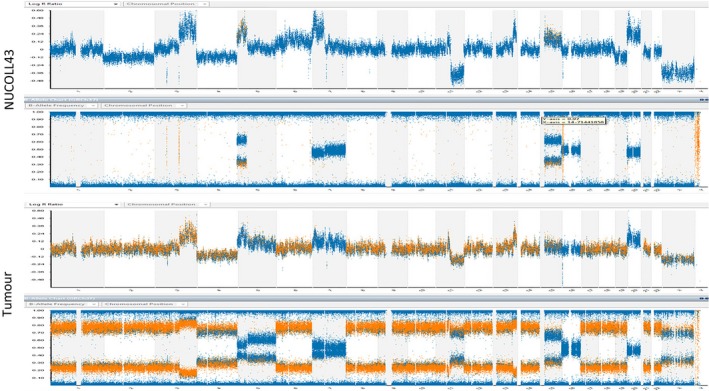
Results of SNP array analysis of NUCOLL43 (upper panels) and tumor (lower panels). The upper chart in each case represents the LogR intensity ratio (copy number), and lower chart represents the B allele frequency (zygosity). The Log R intensity ratios are broadly similar in NUCOLL43 and in tumor. The B allele frequency plots are also similar after subtracting the effect of presumed contaminating normal cells in the tumor

Single nucleotide polymorphism (SNP) array analysis of DNA extracted from NUCOLL43 cells revealed a near‐diploid genome with several segmental chromosome abnormalities (Figure 2). Along with deletion of most of the long arm of chromosome 11 (11q), segmental gains were evident for most of the long of arm of chromosome 3 (3q), the short arm of chromosome 5 (5p) and 7 (7p), and a segment from distal chromosome 13 (13q).

A very high degree of loss of heterozygosity (LOH) was evident, affecting approximately 85% of the genome. Relative losses of whole chromosomes (monosomies) accounted for 25% of this LOH, while 75% corresponds to chromosomal regions present in at least two copies, that is, copy‐neutral LOH *I* uniparental disomy (UPD). Only 15% of the genome had retained allelic heterozygosity.

Chromosome analysis identified a hypodiploid/diploid karyotype, with chromosome counts ranging from 35 to 47. An unusually high degree of cell‐to‐cell karyotypic heterogeneity was recorded, suggesting a derangement of the mitotic segregation process (Figure S2). Structurally abnormal marker chromosomes were present that appear to correspond to the segments of 3q gain, 7p gain and 11q loss.

An almost identical SNP array profile was observed for the original tumor, with copy number and zygosity pattern for chromosomes 1, 3, 4, 8, 9, 10, 11, 12, 13, 14, 15, 16, 18, 20, 21, 22 and X being identical with NUCOLL43, taking into account non‐neoplastic cells in the tumor sample. The segmental imbalances seen on chromosomes 11 and 13 in NUCOLL43 were also present in the tumor. Gains of 5p and 7p were clearly evident in the NUCOLL43 genome: these were much less striking in the primary tumor, suggesting that they were present in only a minority of tumor cells. Analysis of DNA from whole blood from the patient showed no genetic abnormalities.

### 3.3 Proteomics of NUCOLL43 and the original tumor

**Figure 3:**
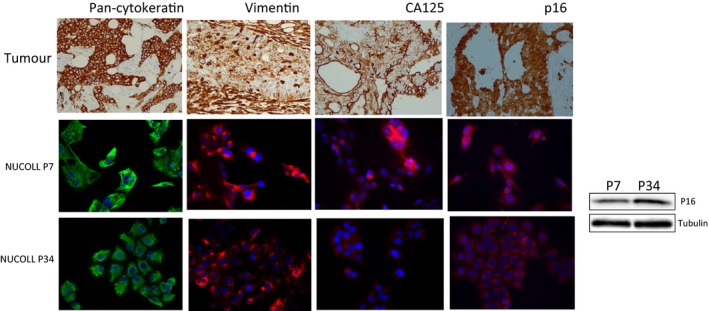
Comparison of protein expression in the original tumor and NUCOLL43 (early and late passage). Both tumor and NUCOLL43 expressed both pan‐cytokeratin and vimentin, indicative of epithelial and mesenchymal characteristics as well as CA125 and p16. Upper panel: pan‐cytokeratin staining (x20); tumor cells show positive cytoplasmic staining. Vimentin staining (x20); tumor cells show patchy positivity, with the stroma surrounding showing strong positive staining. Lower panels: Both passages of NUCOLL43 highly express cytokeratin and vimentin, nuclei counterstained in blue with DAPI. Upper panel: The tumor cells stain positive for CA125 (x20) with clear localization to the cell membrane. Lower panels: CA125 is highly expressed in NUCOLL43 at P7, but low expression seen at P34. Upper panel: The tumor cells are highly positive for P16 (x20) throughout the cell rather than distinctly cytoplasmic or nuclear. Lower panels: Both passages express p16; at P7, the staining is throughout the cell, and at P34, it is localized to the cytoplasm. In NUCOLL43, p16 expression was confirmed by Western blot at both passages

Because of the striking genomic similarity between NUCOLL43 and the original tumor from which it was derived we investigated the phenotypic similarity in terms of expressed proteins. The tumor was positive for pan‐cytokeratin (an epithelial marker), p16 and CA125 (a marker of ovarian cancer) with patchy/focal positive staining for vimentin (a mesenchymal marker) (Figure 3); and negative (null) for p53 (Figure S4) and estrogen receptor (ER) (not shown). Immunofluorescence (IF) analysis showed good concordance with the original tumor with NUCOLL43 positive for vimentin and pan‐cytokeratin at early and late passage. CA125, was expressed in both the tumor and NUCOLL43, but appeared to be weaker at the later passage. P16 was expressed at both passages of NUCOLL43, again correlating with the original histology; however, the pattern of staining differed between the two passages with detection seen throughout the cytoplasm and nucleus at P7, in comparison with the clear cytoplasmic staining seen at P34 cells. In addition to the antigens described here, the original tumor was positive for CKC, CK7 and CK 5/6, negative for GATA3, CDX2, ERα, CK20,p63, AFP, CA19.9, TTF1 and PAX 8 and with patchy/focal staining for calretinin, CD10, RCC, BerEP4 and WT‐1 (data not shown).

**Figure 4:**
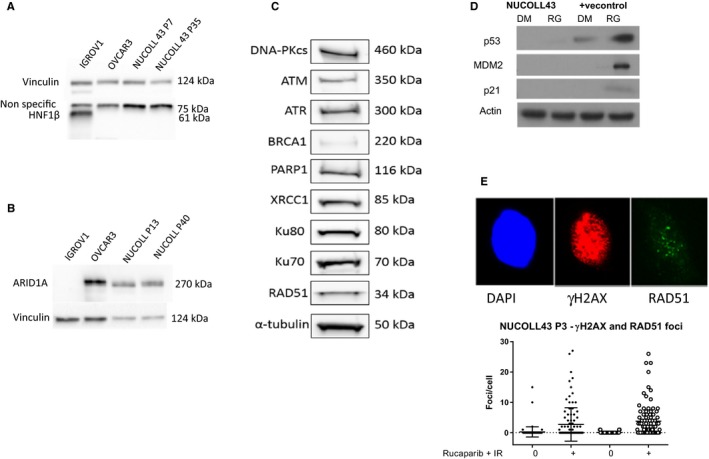
Expression of HNF1β, ARID1A and DNA damage response (DDR) protein expression and activity Early and late passage NUCOLL43 cells were negative for HNF1β protein expression (A). The antibody detected a strong band at 75 kDa that was revealed as being nonspecific by comparison with a positive control (IGROV1) and negative control (OVCAR3). Only in the known positive IGROV1 cells was a band of the correct molecular weight (~61 kDa) observed. The loading control was vinculin (A). Early and late passage NUCOLL43 cells expressed ARID1A by comparison with a negative control (IGROV1) and positive control (OVCAR3) cells; the loading control was vinculin (B). NUCOLL43 cells expressed NHEJ proteins DNA‐PKcs, Ku70 and Ku80, BER proteins PARP1 and XRCC1, HRR proteins BRCA1 and RAD51, and DNA damage sensors ATM and ATR; the loading control was α‐tubulin (C). NUCOLL43 were null for p53 and its targets MDM2 and p21 by Western blot following induction and stabilization by the MDM2 antagonist RG7388 (RG: 0.5 μmol/L 6 h) in comparison with DMSO control (DM) a known p53 wt cell was included as a positive control and actin was the loading control (D). NUCOLL43 (p3) have functional HRR as demonstrated by the formation of RAD51 foci following exposure to 10 μmol/L rucaparib, and the image shows a single nucleus stained with DAPI, yH2AX immunofluorescence, and RAD51 immunofluorescence. The scatter plot shows pooled data from >100 nuclei of the number of yH2AX and RAD51 foci following exposure to rucaparib and 2 Gy radiation vs untreated controls (E)

ARID1A and HNF‐1β have been proposed as novel O‐CCC biomarkers. NUCOLL cells were negative for HNF‐1β (Figure 4A) as no band of the correct molecular weight was seen in NUCOLL43 cells or the negative control cells (OVCAR3) but was clearly visible in the positive control (IGROV1 cells). OVCAR3 as well as IGROV1 cells were positive for HNF‐1β by immunofluorescence (Figure S3). Because of this nonspecific staining, we did not investigate HNF‐1β in NUCOLL43 by immunofluorescence. NUCOLL43 was positive for ARID1A, with OVCAR3 and GROV1 providing positive and negative controls (Figure 4B).

### 3.4 DNA damage response profiling of NUCOLL43

The acquisition of genomic instability is a key event in tumorigenesis and frequently the result of dysregulation of the DNA damage response (DDR).^16^ To investigate the DDR status of NUCOLL43 cells, both the expression of key DDR proteins by Western blot and functional assays were performed. NUCOLL43 cells expressed XRCC1 and PARP1, key components of DNA base‐excision repair that deals with the most common type of DNA damage. DNA double‐strand breaks (DSBs) are highly lethal and are repaired by nonhomologous end joining (NHEJ) and homologous recombination DNA repair (HRR). NUCOLL43 cells expressed the NHEJ proteins DNA‐PKcs, Ku70 and Ku80, as well as the HRR proteins RAD51 and BRCA1. NUCOLL43 cells also expressed ATM and ATR, the major signaling kinases that signal DNA damage, particularly DNA double‐strand breaks to cell cycle checkpoints and DNA repair (Figure 4C).

There was no detectable expression of the tumor suppressor, p53, in the original tumor despite no evidence of chromosome 17p deletion (p53 null: Figure S4A). NUCOLL43 cells were also consistently negative for p53 by Western blot. The MDM2 inhibitor (RG7388) increased p53 stabilization and expression of its downstream targets, p21 or MDM2 in a known *TP53*‐wt cell line (positive control) but failed to increase p53 stabilization and the expression of p21 or MDM2 in NUCOLL43 cells (Figure 4D). RG7388 did not induce p53‐specific changes in gene expression either (Figure S4B) and neither RG7388 nor Nutlin‐3a had any impact on NUCOLL43 cell proliferation (Figure S4C) confirming lack of p53 function.

To confirm that the expression of BRCA1 and RAD51 conferred HRR function, the ability of NUCOLL43 cells to form RAD51 foci in response to DNA damage was assessed. Compared to the untreated controls, rucaparib with IR treatment caused a 10‐fold increase in yH2AX foci) with a 60‐fold increase in RAD51 foci (Figure 4E), indicating that NUCOLL43 has functional HRR.

### 3.5 Drug sensitivity

**Figure 5:**
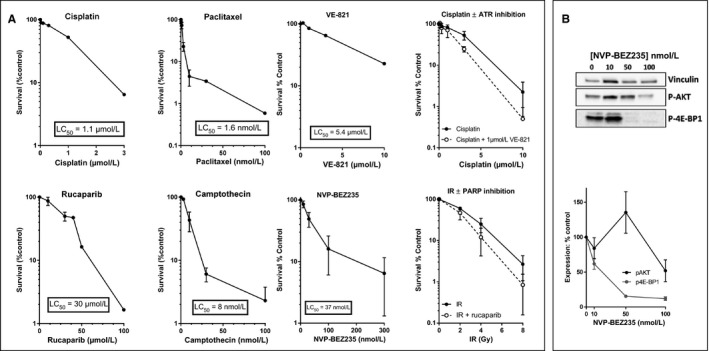
Sensitivity of NUCOLL43 to cytotoxic agents. Results from colony formation assays and cell survival values obtained by normalization of treated cells to untreated controls (A). Error bars show the standard error of the mean (SEM). Data are representative of three independent experiments. The effect of NVP‐BEZ235 on PI3K and mTOR activity was determined by Western blot measuring AKT and 4E‐BP1 phosphorylation, a single representative image is shown, and data normalized to vinculin loading control are shown for three independent experiments (B)

The accumulated data indicated that NUCOLL43 cells were largely similar to the original tumor; therefore, we assessed the sensitivity of NUCOLL43 to drugs commonly used in the treatment of ovarian cancer. We performed colony formation assays, the most direct measure of cell killing (Figure 5A) to calculate the LC_50_ of NUCOLL43 in response to cisplatin (a DNA cross‐linking agent LC_50 _= 1.1 μmol/L), paclitaxel (an antitubulin agent LC_50 _= 1.6 nmol/L), camptothecin (a topoisomerase I poison LC_50 _= 8 nmol/L), rucaparib (a PARP inhibitor LC_50 _= 30 μmol/L), VE‐821 (an ATR inhibitor LC_50 _= 5.4 μmol/L) and NVP‐BEZ235 (a pan‐PI3K inhibitor LC_50 _= 37 nmol/L) (Figure 4). NVP‐BEZ 235 inhibition of PI3‐kinase and mTOR was determined by measuring inhibition of AKT and 4E‐BP1 phosphorylation, respectively, and appeared to be more potent against mTOR than PI3K in NUCOLL43 cells (Figure 5B). Radiosensitization by rucaparib was 1.4‐fold at the LC_50_ and sensitization of cisplatin by VE‐821 was 1.8‐fold at the LC_50_.

## 4 DISCUSSION

We report here the characterization of a cell line derived from a culture of ascites cells from a patient with clear cell ovarian cancer. The cells began to proliferate without artificial immortalization procedures and showed remarkable pan‐genomic similarity to the original tumor with only trivial changes observed. However, the copy number gain of 7p and 5p observed in NUCOLL43 was less striking in the tumor than in the immortalized cells, suggesting that NUCOLL43 was derived by expansion of a clonal sideline within the tumor. Like the original tumor, NUCOLL43 expressed both epithelial (pan‐cytokeratin) and mesenchymal (vimentin) characteristics. In the tumor, vimentin expression was focal/patchy but it was expressed throughout the NUCOLL43 cells, again consistent with the cells being derived from a clonal sideline of the original tumor that was vimentin‐positive and with chromosome 5q and 7p copy number changes. NUCOLL43 has both epithelial and mesenchymal properties. Cells with this phenotype are reported to be particularly tumorigenic due to high invasiveness and motility, with subpopulations of vimentin‐positive cells in ovarian cancer ascitic fluid reported to show greater invasiveness in vitro.^17^ Both the original tumor and NUCOLL cells expressed p16, which is considered a tumor suppressor because it inhibits the initiation of S‐phase. Its upregulation in certain advanced cancers would seem to be somewhat paradoxical. However, increased p16 expression indicates dysregulation of the pRb pathway, and in several tumor types, p16 overexpression is associated with poor prognosis. 18

CA125 serum levels are currently used as a diagnostic indicator of ovarian cancer but levels are highly variable within ovarian cancer, with some tumors showing no increase.^19^, ^20^ In keeping with the CA125 expression in the original tumor, NUCOLL43 cells expressed CA125; however, this seemed to be less pronounced at passage 34 than at passage 7. CA125 is a type I transmembrane glycoprotein, which is shed from the cell surface during proteolytic degradation 21 and studies have shown that trypsin can cleave CA125 from the cell surface.^22^ Recovery times for the reappearance of cell surface markers after trypsinization can vary from 8 to more than 24 hours.^23^ The possibility exists that CA125 levels were lower in passage 34 cells merely because of different recovery from trypsin digestion following routine passage. HNF‐1β expression and ARID1A loss have been suggested as a biomarker for O‐CCC, although its role in tumor progression remains largely unknown.^24^ NUCOLL43 were negative for HNF‐1β but positive for ARID1A by Western blot. The expression of p53 was not detected in the original tumor or in NUCOLL43 cells and lack of p53 function was conclusively demonstrated using MDM2 inhibitors, which neither induced accumulation of p53 or its transcriptional targets nor caused cytotoxicity in NUCOLL43 cells. Mutations and/or deletions of p53 are less frequent in O‐CCC than in HGSOC.^8^ In a study of 155 O‐CCC, 17 showed some unusual morphological characteristics, in addition to the characteristic O‐CCC pathology, and of these 60% were negative for p53 and HNF1β.^25^ NUCOLL43 therefore represents a less frequent subtype of O‐CCC.

Apart from the loss of p53 NUCOLL43 cells expressed all the DDR proteins investigated and, for BRCA1 and RAD51 at least, this was associated with functional DNA repair. The functioning of the HRR pathway underlies their insensitivity to PARP inhibitor‐induced cytotoxicity (LC_50 _= 30 μmol/L). In a study of a panel of human cancer cell lines treated in a similar manner to rucaparib, cells that lacked HRR function had an LC_50_ of substantially less than 10 μmol/L.^26^ HRR dysfunction is also a determinant of sensitivity to cisplatin and the HRR competence of NUCOLL43 cells is consistent with them not being particularly sensitive to cisplatin. In contrast, they appeared to be relatively sensitive to both the topoisomerase I poison, camptothecin, and the antitubulin agent, paclitaxel. Studies of other O‐CCC cell lines have also observed sensitivity to topoisomerase 1 poisons.^27^, ^28^, ^29^ Promising results have been seen in some, but not all, clinical trials of topoisomerase I poisons in O‐CCC^30^ suggesting that topoisomerase I poisons may be effective in the subtype of O‐CCC represented by NUCOLL43. It is not possible to make a direct comparison in terms of LC_50_ values with many of those reported in other studies due to use of different treatment protocols and analysis methods. NUCOLL43 cells were slightly less sensitive to the ATR inhibitor VE‐821 as a single agent than breast cancer cells using a similar assay protocol^31^ and sensitization of cisplatin by VE‐821 appeared to be less than has been reported for other cell lines.^32^ Although NUCOLL43 cells were not sensitive to rucaparib as a single agent the radiopotentiation by rucaparib was about 1.8‐fold in NUCOLL43 cells, which is higher than we have observed in other cell lines using colony formation assays and similar exposure periods.^33^ Interestingly, NUCOLL43 cells were very sensitive to NVP‐BEZ235 in comparison with other cell lines we have investigated.^34^ The PIK3CA‐AKT‐mTOR pathway is emerging as a possible therapeutic target for O‐CCC^35^ with a number of both PIK3CA inhibitors and mTOR inhibitors currently in clinical trials.^36^ A recent paper by Oishi et al^37^ showed that O‐CCC cell lines are more sensitive to NVP‐BEZ235 than HGSOC cell lines; however, the PIK3CA mutation status of the O‐CCC tumor did not appear to impact the sensitivity of these cell lines to NVP‐BEZ235. In our study, NVP‐BEZ235 appeared to be a more potent mTOR than PI3K inhibitor, which may explain why PI3K mutation status in the Oishi et al study did not affect sensitivity.

However, perhaps the most interesting feature of NUCOLL43 is its extremely high level of UPD. High levels of UPD (7.8‐99.9%, median 19%) have been noted in ovarian cancer associated with BRCA mutations with much lower levels (0‐23.2% median = 0.7%) in BRCA wild‐type ovarian cancer.^38^ If UPD is an indicator of HRR defects, it is possible that the tumor from which NUCOLL43 cells were derived had an HRR defect at some stage of its evolution. Potentially, this function needed to be restored to ensure the survival and progression of the tumor, thereby leaving the “genomic scar” associated with its HRR‐defective history. The finding of high UPD in the presence of HRR function indicates that UPD is not a good biomarker for defective HRR.

We believe NUCOLL43 cells represent a subtype of O‐CCC that shows a high level of genomic and phenotypic similarity with the original tumor and will be a useful tool for the investigation of novel therapeutic options for O‐CCC, which are sorely needed given its poor response to current therapy. This cell line has now been deposited with Ximbio.

## CONFLICT OF INTEREST

The authors have no conflict of interests.

## NOVELTY AND IMPACT

NUCOLL43 is a new, somewhat unusual, ovarian clear cell carcinoma (O‐CCC) cell line with an extremely high level of loss of heterozygosity (>80%), most of which is copy neutral. This and its high level of genomic and proteomic similarity to the original tumor make it an interesting model of O‐CCC. Its sensitivity to drugs commonly used to treat ovarian cancer has been determined, and it has been deposited in a commercial bank.
