# Supplementary figures and images for: Characterization and drug sensitivity of a novel human ovarian clear cell carcinoma cell line genomically and phenotypically similar to the original tumor
Source: Cancer Med. 2018 Aug 14;7(9):4744–54. doi: 10.1002/cam4.1724 (PMC6144150; doi:10.1002/cam4.1724)

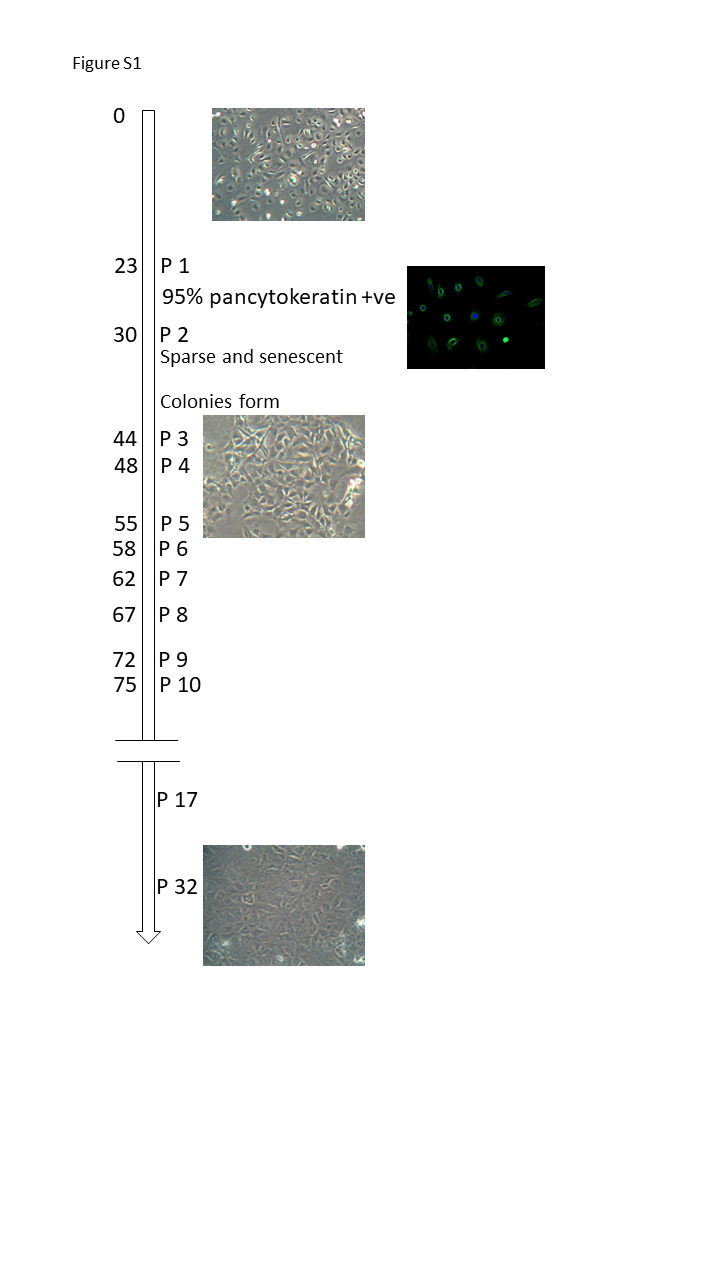

Supplement: Supplementary file 1 [file CAM4-7-4744-s001.tif]

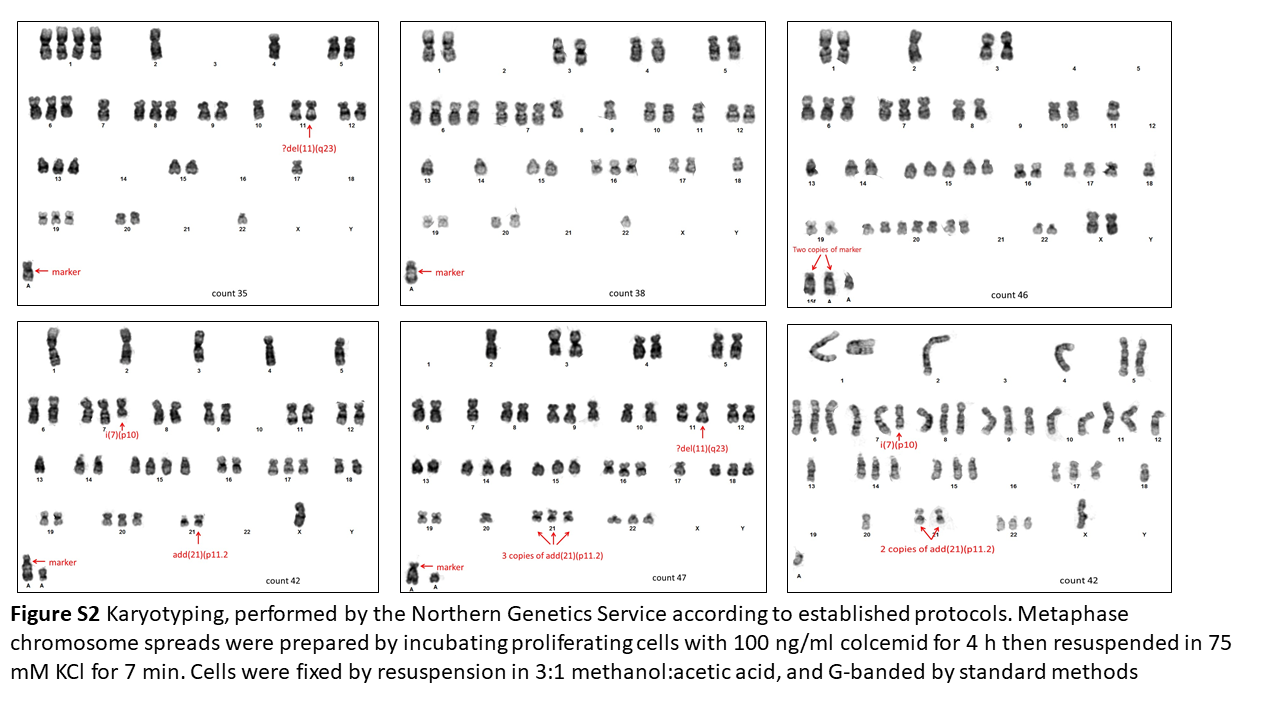

Supplement: Supplementary file 2 [file CAM4-7-4744-s002.tif]

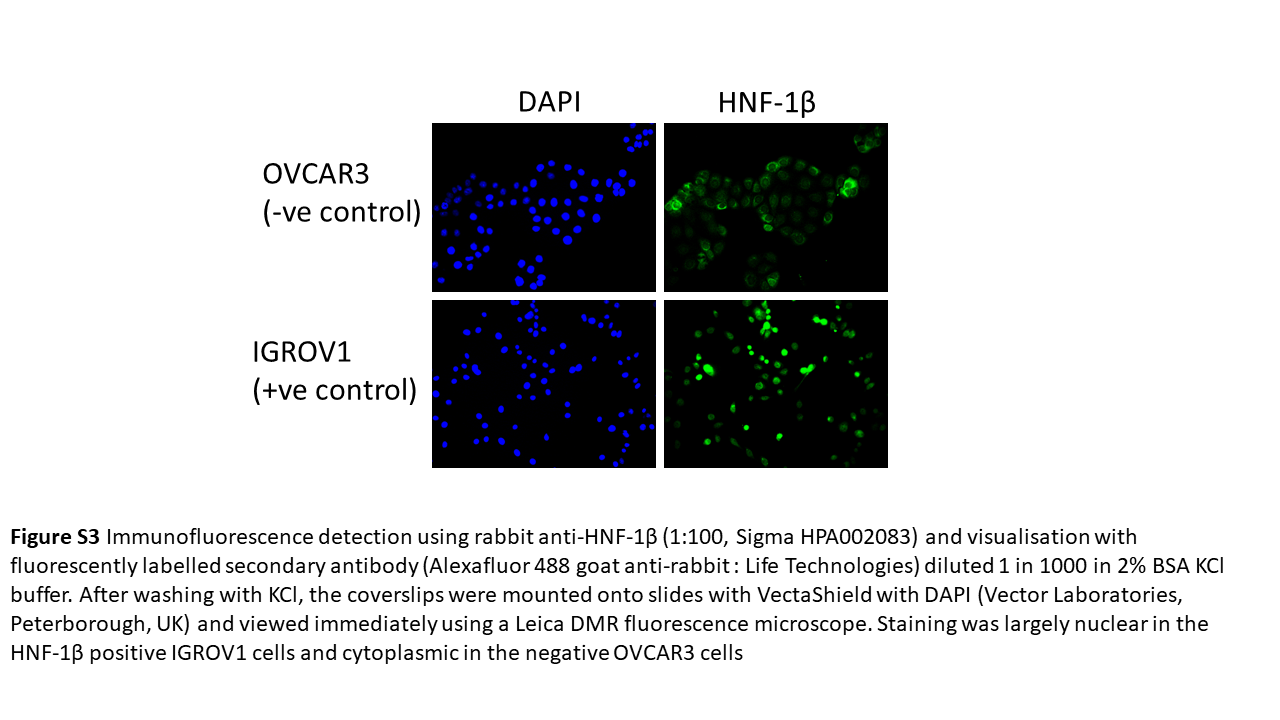

Supplement: Supplementary file 3 [file CAM4-7-4744-s003.tif]

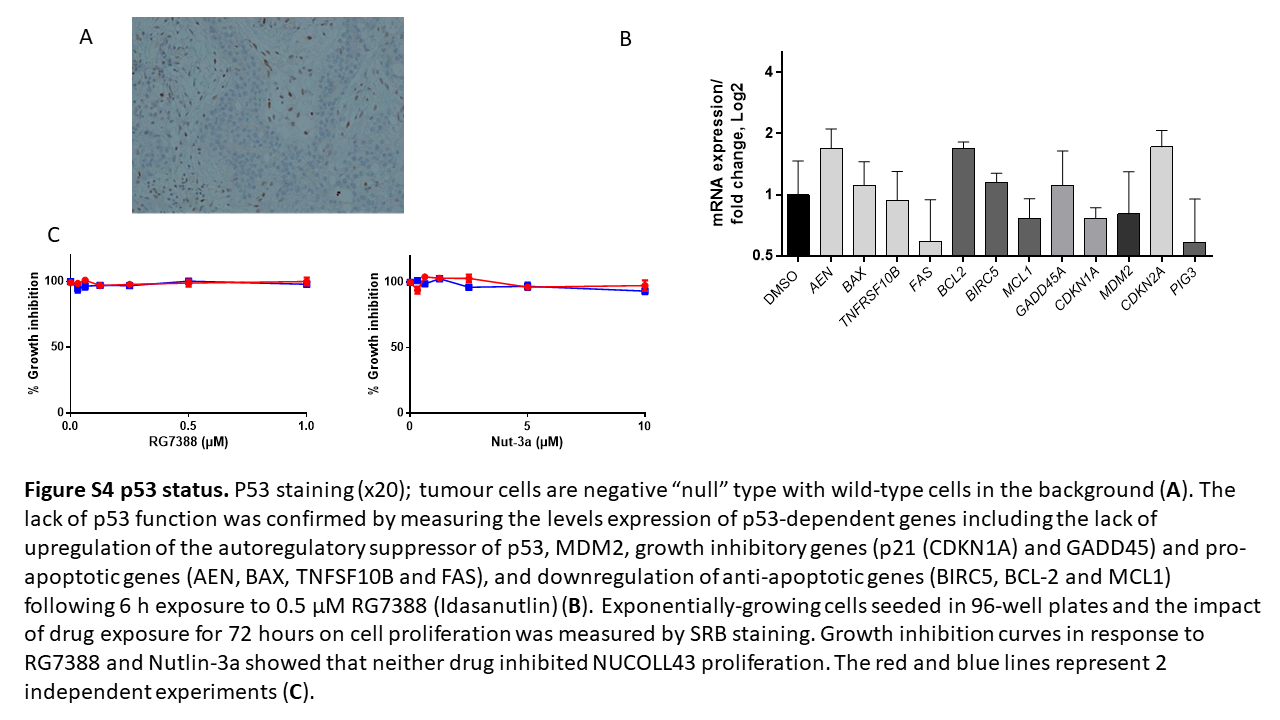

Supplement: Supplementary file 4 [file CAM4-7-4744-s004.tif]
